# A Robotic Context Query-Processing Framework Based on Spatio-Temporal Context Ontology

**DOI:** 10.3390/s18103336

**Published:** 2018-10-05

**Authors:** Seokjun Lee, Incheol Kim

**Affiliations:** Department of Computer Science, Kyonggi University, San 94-6, Yiui-dong, Youngtong-gu, Suwon-si 443-760, Korea; kic@kyonggi.ac.kr

**Keywords:** intelligent service robot, robotic context query, context ontology

## Abstract

Service robots operating in indoor environments should recognize dynamic changes from sensors, such as RGB-depth (RGB-D) cameras, and recall the past context. Therefore, we propose a context query-processing framework, comprising spatio-temporal robotic context query language (ST-RCQL) and a spatio-temporal robotic context query-processing system (ST-RCQP), for service robots. We designed them based on spatio-temporal context ontology. ST-RCQL can query not only the current context knowledge, but also the past. In addition, ST-RCQL includes a variety of time operators and time constants; thus, queries can be written very efficiently. The ST-RCQP is a query-processing system equipped with a perception handler, working memory, and backward reasoner for real-time query-processing. Moreover, ST-RCQP accelerates query-processing speed by building a spatio-temporal index in the working memory, where percepts are stored. Through various qualitative and quantitative experiments, we demonstrate the high efficiency and performance of the proposed context query-processing framework.

## 1. Introduction

Dey [1] defined context knowledge in a broad sense as “any information that can be used to characterize the situation of an entity”. However, for intelligent service robots, the concrete context knowledge of the robot’s domain needs to be redefined. Turner [2] defined context knowledge for intelligent agents into three categories, based on which Bloisi [3] redefined robot-oriented context knowledge: environmental knowledge, task-related knowledge, and self-knowledge. Environmental knowledge includes physical-space information about the environment outside the robot, such as the locations of people and objects and an environmental map. Task-related knowledge includes tasks that can be performed by robots and the constraints of these tasks. Self-knowledge includes the internal conditions of robots, such as joint angle and battery level. According to the definition by Bloisi, the context knowledge of robots includes not only static knowledge, such as common sense, but also dynamic knowledge that has high time dependency as it continuously changes in real time. This context knowledge is indispensable for the generation of robot task plans (task planning) [4,5], human–robot collaboration or multiple robot collaboration [6,7], and context-knowledge-providing services [8,9]. Therefore, the performance and application scope of robots depends on how diverse and complex the context knowledge that the service robot can recognize and understand is.

Among the many different types of context knowledge, this study focused on context knowledge related to the environment, and in particular, on the locational information of individual objects and the three-dimensional (3D) spatial relations among objects in a home environment. The locational information (pose of individual objects) is sub-symbolic knowledge obtained from sensors, such as RGB-depth (RGB-D) cameras, and 3D spatial relations are abstracted symbolic knowledge that must be derived from this locational information. Three-dimensional spatial relations are comprehensive knowledge commonly required in most robot domains and are essential prior knowledge for deriving complex context knowledge, which is more difficult to determine; for example, the intention of external agents [10,11]. However, 3D spatial relations must be tracked in real time because they continuously change time independently of the robot and must be able to store and retrieve past context knowledge.

To examine examples of applications of 3D spatial relations, first, the preconditions of each task must be satisfied for robots to generate a task plan. For example, to generate a task plan for delivering a drink to a person, the following preconditions must be satisfied: a cup is filled with the drink and the robot is holding this cup. In another example, a context-knowledge-providing service must provide various context knowledge requested by the user in an indoor environment. For example, the user requests context knowledge such as “is there orange juice in the refrigerator now?” and “where is the tumbler, which was on the table yesterday, now?” These examples show that in terms of service, the context knowledge ultimately required by a robot is abstracted symbolic knowledge.

A service robot must be able to retrieve context knowledge stored in the working memory anytime or infer abstracted context knowledge when necessary. The retrieval of context knowledge requires a context query language and processing method for accessing the working memory inside the robot and manipulating knowledge. The important requirements of a context query language can be found in [12,13]. Typically, context knowledge is closely related to the physical spatial relations and has high time dependency as it frequently changes over time. Therefore, the context query language must have high expressiveness to query context knowledge of various periods. Furthermore, as the main purpose of context query is to retrieve and infer abstracted symbolic knowledge, the grammatical structure of the query language must be designed to write highly concise and intuitive queries.

As the grammatical structure of a query language is greatly dependent on the knowledge representation model for storing context knowledge, the knowledge representation model must be defined first. The general knowledge representation method of knowledge-based agents, such as service robots, involves the use of description logic-based ontology [14,15,16,17]. Every knowledge in ontology is represented in a statement composed of a subject, a predicate, and an object. Thus, the query language and processing method for retrieving context knowledge depends on this triple format. However, as the triple format can only express one fact, representing when this fact occurs and whether it is valid is difficult. For example, the triple format of “<red_mug> <on> <table>” cannot express that this fact was valid yesterday but is no longer valid today because the red mug has been moved to the shelf.

The existing work on robot context query includes OpenRobots Ontology (ORO) [16], KnowRob [17], and SELECTSCRIPT [18]. ORO provides a knowledge-query API (Application Programming Interface) based on SPARQL (a semantic query language able to retrieve and manipulate data stored in RDF (Resource Description Framework)) [19], which is a semantic Web query language, and ORO can thus retrieve and manipulate knowledge written in RDF (Resource Description Framework). However, ORO does not have a specific knowledge representation method to specify the valid time of knowledge and does not consistently maintain past context knowledge. Hence, ORO is limited in allowing only the current context knowledge to be queried. KnowRob provides prolog query predicates based on first-order logic in accordance with the semantic Web library of SWI-Prolog (a free implementation of the programming language Prolog) [20]. KnowRob provides query predicates for expressing the valid times of perception information obtained from sensors and for querying and inferring the context knowledge of various periods. However, KnowRob does not support time operators and time constants for querying various periods and requires complex, inefficient queries. SELECTSCRIPT is an SQL (Structured Query Language)-inspired declarative query language of the script format. It provides embedded unary and binary operators to enable the context knowledge to be queried. However, as with ORO, SELECTSCRIPT does not have a specific knowledge representation method to specify the valid time of knowledge and does not consistently store past context knowledge. Thus, SELECTSCRIPT is also limited in allowing only the current context knowledge to be queried. For these three works, efficient query-processing methods, such as spatio-temporal indexing, have not been considered.

Against this backdrop, this study proposes the spatio-temporal robotic context query language (ST-RCQL), which allows the query of time-dependent context knowledge of service robots, and the spatio-temporal robotic context query-processing system (ST-RCQP), which allows the real-time query-processing. The ST-RCQL proposed in this paper assumes that the 3D spatial relations among objects are retrieved from the individual spatio-temporal perceptions of indoor environmental objects obtained from RGB-D cameras. However, although this type of assumption is adopted by many spatial query systems, it has a disadvantage in that a very complex query must be written to obtain symbolic knowledge [21,22]. As service robots require more abstract symbolic knowledge, in this study, query grammar was designed to allow very concise and intuitive queries instead of complex queries. For actual query-processing, query translation rules were designed to automatically translate queries to complex queries for internal processing. Furthermore, query grammar was designed to enable queries involving context knowledge of various periods by providing time operators based on Allen’s interval algebra. The query-processing was accelerated using a spatio-temporal index by considering the characteristics of service robots with a real-time property from the perspective of a query-processing system. Furthermore, it is more appropriate for robots to obtain specific context knowledge required at some time point rather than always inferring and accumulating all context knowledge explicitly. Therefore, we adopted a query-processing method based on backward chaining [23]. To verify the suitability of the proposed ST-RCQL as a robot context query language and the efficiency of ST-RCQP, a query-processing system was implemented using SWI-Prolog and JAVA programming language, and the results of qualitative and quantitative experiments using this system are introduced in the following sections.

## 2. Related Works

### 2.1. Repesentation and Storage of Context Knowledge

The OpenCyc [14] provides OWL-DL (a sublanguage of OWL (Web Ontology Language) based on description logic)-based upper ontology that the semantic Web community agrees on. To express knowledge about the specific domain of a robot, ORO [16] and Knowrob [17] expand the upper ontology of OpenCyc in a robot-oriented manner. ORO expands the upper ontology of OpenCyc to represent specific objects and actions that appear in scenarios, such as packing and cleaning a table. The context knowledge stored by ORO comprises spatial relations, including topological, directional, and distance relations, as well as abstracted symbolic knowledge such as visibility of agents and reachability of objects. For storing context knowledge in working memory, the triple store of OpenJena is used. The perception information collected from sensors is used for inferring context knowledge but is not stored in the working memory. For efficient management of working memory, ORO separately stores short-term, episodic, and long-term knowledge. The short-term and episodic knowledge are deleted every 10 seconds and five minutes, respectively, but the long-term knowledge is not deleted. Examples of short-term, episodic, and long-term knowledge are spatial relations, actions, and TBox, which is an ontology schema, respectively.

KnowRob expands the upper ontology of OpenCyc for specific objects, tasks, actions, and perception information, which are observed in scenarios occurring at home, such as making a pancake in the kitchen. The context knowledge stored by KnowRob is mostly perception information, such as the pose and bounding box of objects obtained from sensors. It does not store abstracted symbolic knowledge such as spatial relations. For storing context knowledge in the working memory, rdf_db triple store, which is included in the semantic Web library of SWI-Prolog, is used.

Ontology-based Unified Robot Knowledge (OUR-K) [15] is another context knowledge representation model. OUR-K categorizes knowledge into context, spaces, objects, actions, and features classes and each class has three layers. Among them, the bottom layer inside the context class represents a spatial context, which represents the spatial relations between objects. The spatial context combines with the temporal context in the middle layer and leads to more abstracted contexts.

### 2.2. Context Query Language

ORO provides an API for context query, with *find* as the main function. As a SPARQL engine is present at the backend of the *find* function, context knowledge can be retrieved from the triple pattern by inheriting the expressive power of the SPARQL as it is. However, ORO cannot query past context knowledge because it always stores and maintains only recent spatial relations.

For context query, KnowRob expands the semantic Web library of SWI-Prolog and supports the prolog predicates that can construct queries that include the valid time of knowledge. The main predicate for knowledge retrieval is *holds*; it can retrieve context knowledge valid at a specific time in the present or past. This is because it is possible to enter the context predicate and valid time of the triple pattern as arguments. However, in the *holds* predicate, the valid time can be entered only as a time point and only *at* is supported for time operator, resulting in very complex queries.

Another query language is SELECTSCRIPT [18], which constructs queries for XML (eXtensible Markup Language)-based simulation files that are continuously updated. In particular, SELECTSCRIPT supports embedded binary operators that can query spatial relations in the WHERE clause and supports the function to obtain the results in the form of prolog predicate logic. However, one limitation of SELECTSCRIPT is that it cannot query past context knowledge because it always updates and maintains the simulation file as latest information.

### 2.3. Context Reasoning

From the spatial reasoning and knowledge (SPARK), which is a geometric reasoning module, ORO infers abstracted context knowledge. SPARK infers spatial relations among objects, robots, and the user, as well as visibility and reachability from the perception information obtained from sensors, such as 2D fiducial marker tracking and human skeleton tracking, whenever perception information is inputted from sensors. SPARK is a forward reasoning method that infers context knowledge, delivers the result to the ontology module of ORO and stores it in the working memory. When storing context knowledge in the working memory, it performs consistency checks by using the Pellet reasoner.

KnowRob infers abstracted context knowledge from a spatio-temporal reasoning module developed through SWI-Prolog. This module infers spatial relations between objects from the bounding box and the center of objects obtained from sensors. Unlike the forward reasoning method of ORO, KnowRob employs the backward reasoning method, which infers context knowledge only when requested by a query using a computable predicate.

Another estimator is the QSRlib (a library that allows computation of qualitative spatial relations and calcul) [24], which is a software library implemented using Python, and it can be embedded in various intelligent systems. QSRlib provides geometric reasoning for distance relations, such as qualitative distance calculus [25]; directional relations, such as cardinal direction (CD) [26] and ternary point configuration calculus [27]; and topological relations, such as rectangle algebra [28] and region connection calculus [29], from video information obtained from RGB-D cameras. In addition, it also provides geometric reasoning for the movements of objects such as qualitative trajectory calculus [30].

## 3. Expression and Management of Robot Context Knowledge

### 3.1. Knowledge Representation

**Figure 1:**
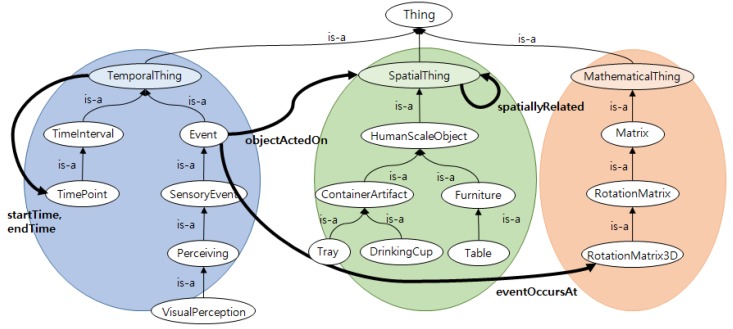
A part of ontology for context knowledge.

As a context query language depends on a knowledge representation model for representing and storing context knowledge, the model must be defined before the context query language is designed. For this purpose, we constructed a context ontology, as shown in Figure 1, in accordance with the standard semantic Web language, RDF/OWL. The RDF/OWL based on descriptive logic (DL) can define facts or knowledge of the triple format for logical reasoning. Figure 1 (left) shows class hierarchy and properties for representing time-related knowledge. Representative classes include the *Event* class for representing the individual spatio-temporal information of objects, such as the *VisualPerception* class, indicating the pose of objects and the *TimePoint*, *TimeInterval* class, indicating the valid time of specific events. As *startTime* and *endTime* properties are defined for the *TemporalThing* class at the top, subclasses, such as *VisualPerception*, can inherit these properties to represent valid time of event.

**Figure 2:**
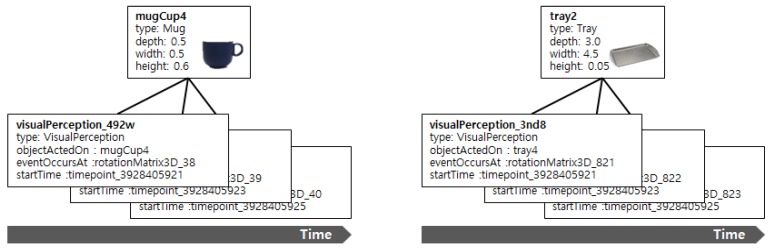
An example of visual perceptions.

Figure 2 shows the visual perception instances of objects created by referring to the ontology in Figure 1. The box above of visual perception instances shows the basic information about each object, such as class type, depth, width, and height, which the service robot knows in advance. Then, when visual perception of the object occurs, the visual perception instances of the object (*visualPerception_492w*, *visualPerception_3nd8, ...*) are created. These instances include information about the class type (*type*), perceived object (*objectActedOn*), perceived pose of object (*eventOccersAt*), and perceived time (*startTime*). This representation method can effectively represent the spatial information of objects according to the valid time and is advantageous for building the spatio-temporal index. The center of Figure 1 shows class hierarchy and properties to represent objects in the indoor environment and the 3D spatial relations. The representative classes include container classes (*ContainerArtifact*), such as *DrinkingGlass*, *Tray* and furniture classes such as *Table* and *Shelf*.

**Figure 3:**
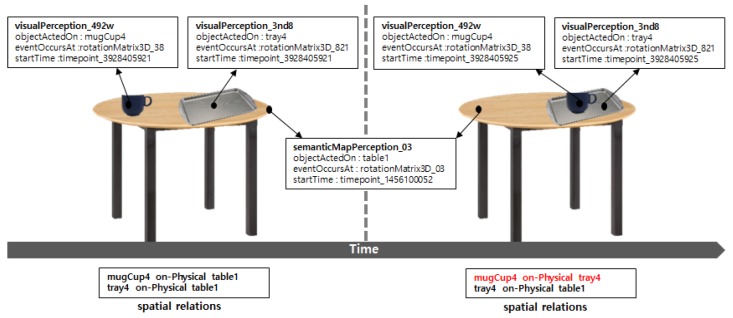
An example of 3D spatial relations between objects.

**Figure 4:**
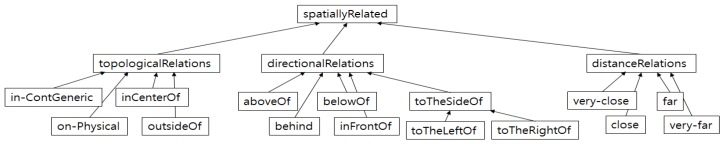
Properties of 3D spatial relation.

Figure 3 shows the 3D spatial relations among the objects created from the ontology in Figure 1. The top part of Figure 3 shows the visual perception instances of the mug cup (*mugCup4*) and tray (*tray4*) perceived at *t1* (*timepoint_3928405921*) and *t2* (*timepoint_3928405925*). The middle part of Figure 3 shows the semantic map instance for the table (*table1*) comprising the visual perception information. The representation method for the semantic map instances is similar to that of visual perception instances. However, the objects that belong to semantic map instances are assumed to be always stationary. The bottom part of Figure 3 shows expressions of 3D spatial relations among the objects: “The mug cup is on the table (*mugCup4 on-Physical table1*)” and “The tray is on the table (*tray4 on-Physical table1*).” These spatial relations are inferred from poses and geometries in the visual perception instances and the semantic map instances. The spatial relations of the mug cup and furniture, which are the result of inference, implicitly include time dependency on *t1* and *t2*, which are time points when the poses of the mug cup were perceived. For example, the valid time of the mug cup on the table is *t1*, and the valid time of the mug cup on the tray is *t2*. The properties of spatial relation considered in this study are shown in Figure 4.

Figure 4 shows the sub-properties of the *spatiallyRelated* property for representing the 3D spatial relations in Figure 1. The properties of spatial relation are largely divided into topological, directional and distance relations. The topological relation properties include *on-Physical*, *in-ContGeneric*, *in-CenterOf*, and *outsideOf*, whereas the directional relation properties include *toTheLeftOf*, *inFrontOf-Generally*, and *aboveOf-Generally*. Finally, Distance relation properties include *very-close, close, far, and very-far*.

### 3.2. Knowledge Management

This study followed the RDF/OWL triple format for unified representation and smooth sharing of context knowledge; however, the knowledge is internally stored after being translated into prolog facts based on first-order logic. This is because of the geometric reasoning to infer 3D spatial relations from the positions of individual objects. Prolog is an advanced logic programming language that enables ontology-based logical inference according to description logic, horn logic, etc., and enables geometric reasoning based on arithmetic operations.

**Figure 5:**
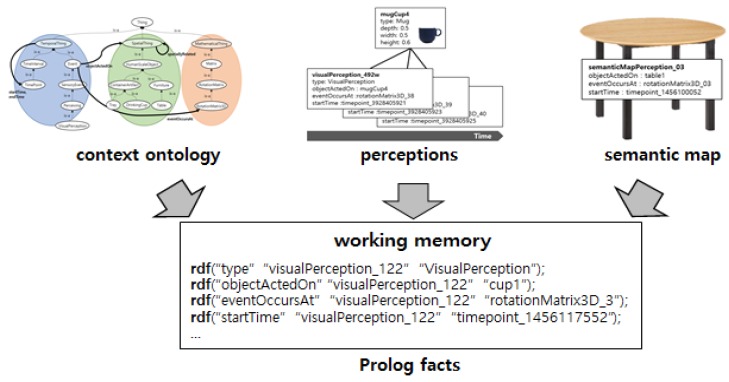
Translating context knowledge in the form of RDF/OWL to Prolog facts.

**Figure 6:**
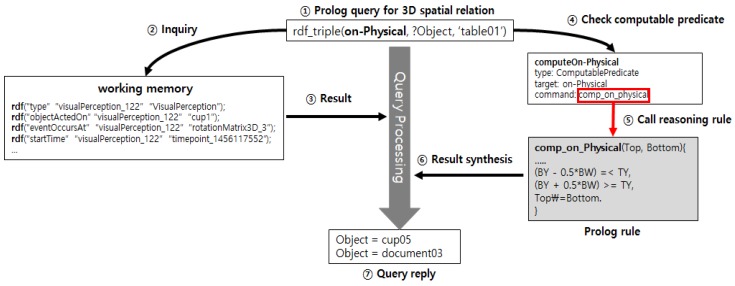
Backward reasoning using computable predicate.

As shown in Figure 5, the context knowledge stored inside the robot follows the static context ontology, which exists in the ontology file format, and represents dynamic visual perception instances, which are perceived in real time through the RGB-D camera, and semantic map instances, which are made in advance. The context ontology and semantic map instances are loaded into the working memory when the robot is started, and the perception information is newly stored at each perception. This study employed the backward reasoning method to present the 3D spatial relations among objects only as responses to query requests without storing them in the working memory. Considering the hardware limit, real-time property, etc., of the robot, obtaining only the context knowledge specifically demanded at a specific time point is more appropriate for the robot than to identify and accumulate all abstracted context knowledge whenever perception information is input. The backward reasoning adopted in this study is illustrated in Figure 6.

Figure 6 shows the backward reasoning method using a computable predicate [13]. As shown in this figure, when a Prolog query (*rdf_triple (‘on-Physical’, ?Object, ‘table01′)*) is input for 3D spatial relations, it retrieves working memory, and then checks whether on-Physical, which is the predicate of the query, is registered as the computable predicate in the context knowledge ontology. If the predicate of the query is not registered as a computable predicate, the query is processed by just retrieving the working memory through the triple pattern. However, if it is registered, not only is it retrieved, but the built-in reasoning rule is also invoked, and the result is included in the response to the query.

## 4. Design of Robot Context Query Language

**Figure 7:**
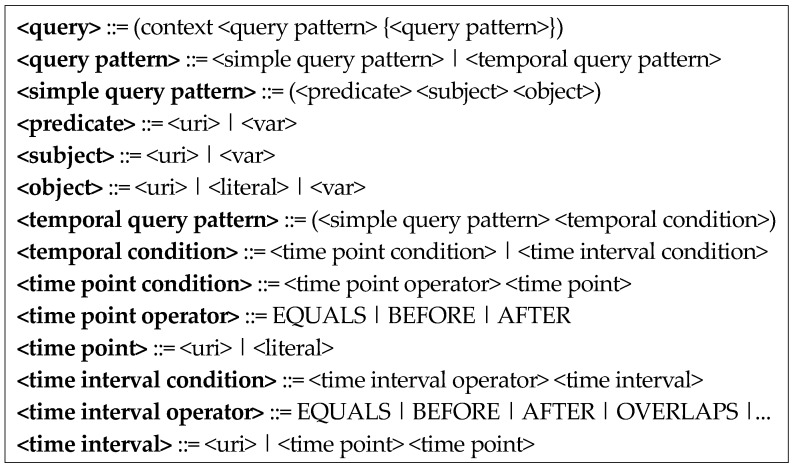
Grammar structure of the context query language.

As the 3D spatial relations change continuously over time, a context query language with time dependency is required. Furthermore, as the context knowledge mainly required of robots in terms of service is abstracted symbolic knowledge, such as 3D spatial relations rather than low-level values, such as object poses, the context query language must be written very concisely and intuitively. To satisfy these requirements of the context query language, we propose the grammatical structure of the context query language in Figure 7; this structure is written in the extended Backus Naur form.

**Figure 8:**
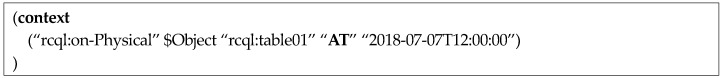
An example of a context query using a time-point operator.

**Figure 9:**
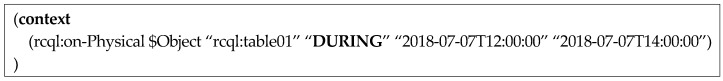
An example of a context query using a time-interval operator.

**Figure 10:**
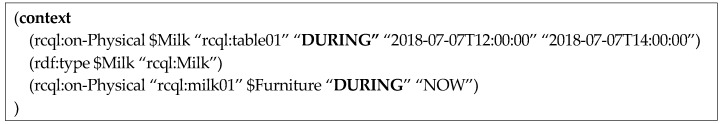
An example of a multiple context query using time-interval operators.

This grammatical structure is interpreted as follows. A query is a repetition of a query pattern, which is either a simple or temporal query pattern. A simple query pattern is a triple format consisting of a predicate, a subject, and an object. The predicate and subject are a URI (uniform resource identifier) or variable, and the object is a URI, literal, or variable. The temporal query pattern is composed of a simple query pattern and a temporal condition, which is composed of a time-point condition and a time-interval condition. The time point condition is composed of a time-point operator and a time point. The time-point operators include EQUALS, BEFORE, and AFTER, while the time point is a URI or literal. The time-interval condition is composed of a time-interval operator and a time interval. Thirteen time-interval operators are present and are based on Allen’s theory; these include EQUALS, BEFORE, AFTER, and OVERLAPS. The time interval is a URI or consists of two time points. The grammatical structure of the context query language in Figure 7 can be used to write context queries, as shown in Figure 8, Figure 9 and Figure 10. Figure 8 shows a context query using a time-point operator.

Figure 8 represents the query “What is the object on the table at 12:00?” In this example, the first three elements after the header *context* are a predicate (*rcql:on-Physical*), a subject *($Object*), and an object (*rcql:table01*), respectively. Here, the subject is a variable. The fourth element is the time-point operator EQUAL and the last fifth element is the time literal value (*2018-07-07T12:00:00*), which is the operand of the time-point operator. Figure 9 illustrates a context query using a time-interval operator.

Figure 9 represents the query, “What is the object that was on the table between 12:00 and 14:00?” The fourth element in this query is the time-interval operator DURING and the fifth and sixth elements represent the start time (*2018-07-07T12:00:00*) and end time (*2018-07-07T14:00:00*), respectively, for the operand of the time-interval operator. Finally, Figure 10 illustrates a multiple context query using time-interval operators.

As shown in Figure 10, a multiple context query refers to a query consisting of two or more query patterns. The query in this figure represents “Where is the milk that was on the table during lunch between 12:00 and 14:00 now?” In this query, the first query pattern queries about the objects that were on the table during lunch by using a time-interval operator. The second query pattern only queries about the milk among the objects queried in the first query pattern. The last query pattern queries about the furniture on which the milk (queried about in the second query pattern), is placed presently. Here, NOW is the time constant that dynamically receives the current time.

The query languages of ORO and SELECTSCRIPT can only query about the current context knowledge because the valid time of context knowledge cannot be specified. However, ST-RCQL can query context knowledge that is valid at specific times both in the past and present. Similarly, KnowRob allows the query of a valid past context knowledge. However, KnowRob provides only one time-operator, whereas ST-RCQL provides a rich set of 13 time-operators following the Allen’s interval theory, thus allowing very efficient queries of context knowledge in different periods. Furthermore, more concise and abstracted queries are possible as ST-RCQL supports time constants such as NOW and TODAY.

## 5. Robot Context Query-processing System

### 5.1. System Structure

**Figure 11:**
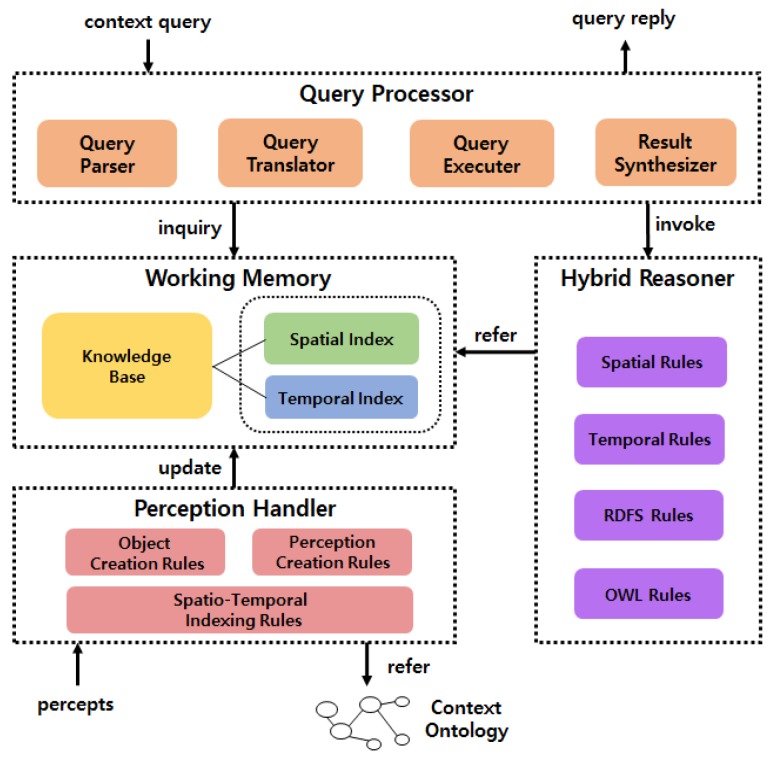
Structure of context query-processing system.

The core context query-processing abilities required in this study are to retrieve the context knowledge of the valid time satisfying the time operator and the inference of 3D spatial relations among objects from poses of the individual object. Therefore, in this study, context query language was translated to Prolog queries, which were then processed. Arithmetic operations, such as geometric operations, can be included in the reasoning rules because the advanced logic programming language, Prolog, programs procedural languages, such as C, by using a logical language. Furthermore, to quickly process queries of service robots working in real time, spatio-temporal indices were built in the working memory and referenced to accelerate query-processing by increasing the knowledge access and reasoning speeds. Figure 11 shows the structure of the context query-processing system that meets these requirements.

In the context query-processing system, the perception handler stores the visual perception information (percepts) received in the working memory, which is an internal storage, in real time. When storing the visual perception information in the working memory, the system refers to the context ontology and dynamically updates the spatio-temporal indices. When a context query is inputted in this situation, the query processor translates the context query into a Prolog query and retrieves context knowledge in the working memory or derives the result of the query by using the hybrid reasoner. Then, it replies the final result to the query by synthesizing these results.

### 5.2. Spatio-Temporal Index

**Figure 12:**
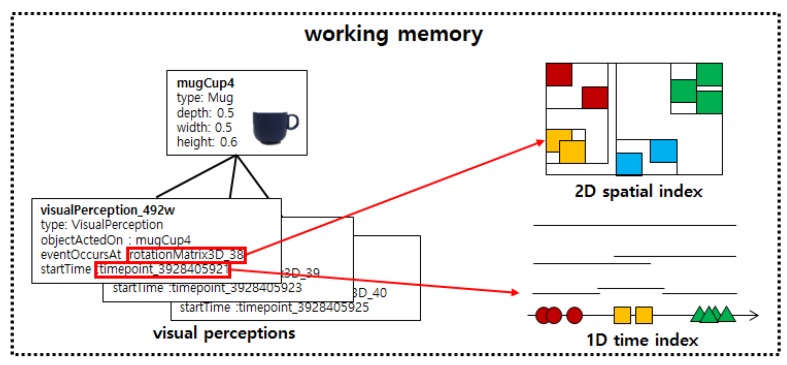
Building of spatio-temporal index for visual perception instances.

For the real-time property of the service robot, the 3D spatial relations must be quickly inferred from the visual perception instances of the objects inputted to the working memory at a rate of 10 frames per second. The largest costs in this process are the costs of accessing the visual perception instances and inferring 3D spatial relations from them. In this study, to reduce the cost of accessing the visual perception instances, as shown in Figure 12, a time index was built from the valid times of the visual perception instances, and a spatial index was built from the poses of objects to reduce the cost of inferring 3D spatial relations.

The time index in Figure 12 was built by using a 1D R-tree [31]. This time index is referenced when visual perception instances satisfying the time operator are accessed. The spatial index was built by using the 2D R*-tree [32]. Although the pose of object is 3D spatial information, the spatial index was built in a 2D R*-tree because the 3D plane viewed from the top can sufficiently represent the locality of the objects in an indoor environment. Furthermore, it is much faster to update a 2D spatial index than a 3D spatial index when considering the cost of updating the spatial index whenever the poses of objects is inputted in real time.

### 5.3. Translation of Context Query

In this study, the query grammar was designed to enable very concise and intuitive context queries to be written for retrieving abstracted symbolic knowledge. However, to process actual queries that include time operators and geometric operations, such as 3D spatial reasoning, the inputted context query must be translated into a Prolog query for internal processing. Accordingly, the query translation rules were designed, as shown in Table 1.

The query translation rules in Table 1 are divided into simple spatial queries not comprising time operators, spatio-temporal queries comprising time-point operators, and spatio-temporal queries comprising time-interval operators. First, for the context query translation rule for queries not comprising time operators, the most recent visual perception information of objects was retrieved and the query was translated into a Prolog query for verifying the spatial relation predicate from the poses of objects.

**Figure 13:**
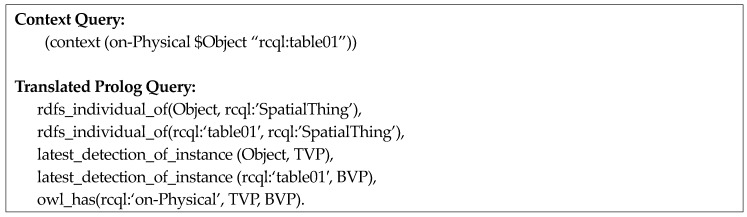
Illustration of translation for simple spatial query pattern. *BVP* is the visual perceptions of base object (*table01*) and *TVP* is the visual perceptions of target objects (not include *table01*).

For example, if the context query, “What is the object on *table01*?” in Figure 13 is inputted, the semantic map instance of *table01* and the most recent visual perception instances of other objects is retrieved, and the spatial relation predicate between the poses of *table01* (*BVP* in Figure 13) and the other objects (*TVP* in Figure 13) is verified, while the remaining objects satisfying this query are returned as the result.

Next, the query comprising a time-point operator retrieves visual perception instances of the period satisfying the time-point operator, and the query is translated into a Prolog query for verifying the spatial-relation predicate from the poses of these objects.

**Figure 14:**
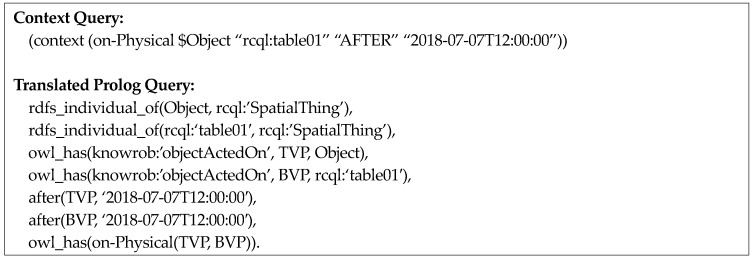
Illustration of translation for spatio-temporal query pattern one.

For example, when the context query “What is the object on *table01* after 12:00 p.m. on 7 July 2018?” is inputted, as shown in Figure 14, the visual perception information of *table01* and other objects after 12:00 p.m. on 7 July 2018 is retried. Then, the spatial relation predicate *on-Physical* between the pose of *table01* (*BVP* in Figure 14) and poses of other objects (*TVP* in Figure 14) is verified and the objects satisfying this condition are retrieved again.

Finally, the query including a time-interval operator retrieves visual perception information of the period that satisfies the time-interval operator and is translated into a Prolog query for verifying the spatial relation predicate from these poses of objects.

**Figure 15:**
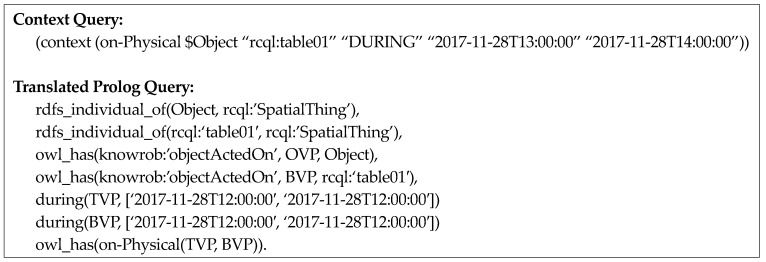
Illustration of translation for spatio-temporal query pattern two.

For example, as shown in Figure 15, if the context query “What is the object on *table01* between 12:00 p.m. and 14:00 p.m. on 28 November 2017?” is inputted, the semantic map instance of *table01* and visual perception instances of other objects whose valid time is between 12:00 p.m. and 14:00 p.m. on 28 November 2017 is queried first. Then, the spatial-relation predicate between the pose of *table01* and poses of other objects is verified and the objects that satisfy this condition are retrieved again.

## 6. Implementation and Experiment

### 6.1. Implementation

To analyze the performance of the context query language (ST-RCQL) and the query-processing system (ST-RCQP) proposed in this study, ST-RCQP was implemented as follows.

ST-RCQP was implemented using Java programming language in the environment of Windows 10 on a 64-bit i5-6600 CPU. In particular, to implement the Prolog-based reasoning engine and working memory inside the system, the Semantic Web Library 3.0 package of SWI-Prolog [20] was used, and the Space package of SWI-Prolog was used to implement indices in the working memory. In addition, the JPL library was used as the bidirectional interface between Java and Prolog.

### 6.2. Experiment

The experiments on performance analysis were largely divided into qualitative and quantitative. First, the qualitative experiment was conducted to prove the high expressive power of ST-RCQL. The experimental method involved writing queries by using ORO, KnowRob, SELECTSCRIPT, and ST-RCQL to obtain answers for three spatio-temporal contexts and compare the queries.

**Figure 16:**
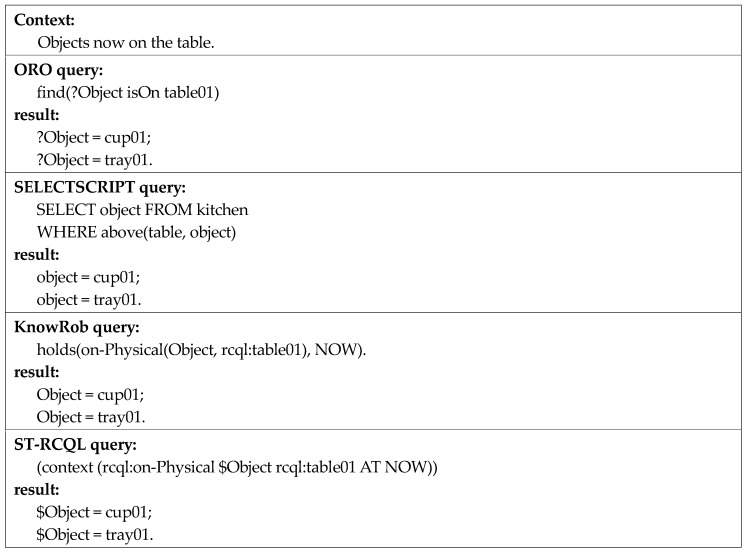
Comparison of spatio-temporal context queries one.

**Figure 17:**
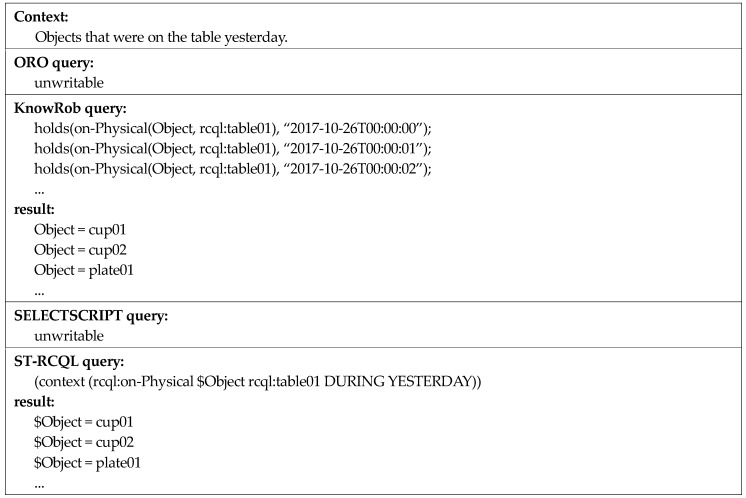
Comparison of spatio-temporal context queries two.

The first context is “objects now on the table.” This context includes the temporal context of “now” with the spatial context of “on”. According to Figure 16, all the four query languages have the expressive power to query this spatio-temporal context. Every query includes a time operator or spatial predicate for the spatio-temporal query. In the case of ORO and SELECTSCRIPT, the query only includes a spatial predicate with no time operator. ORO and SELECTSCRIPT do not store past context knowledge but continuously update and maintain the current spatial context knowledge. Thus, their queries for the first context are valid. However, when writing a query for a past context, the occurrence of the problem shown in the second context in Figure 17 cannot be avoided.

**Figure 18:**
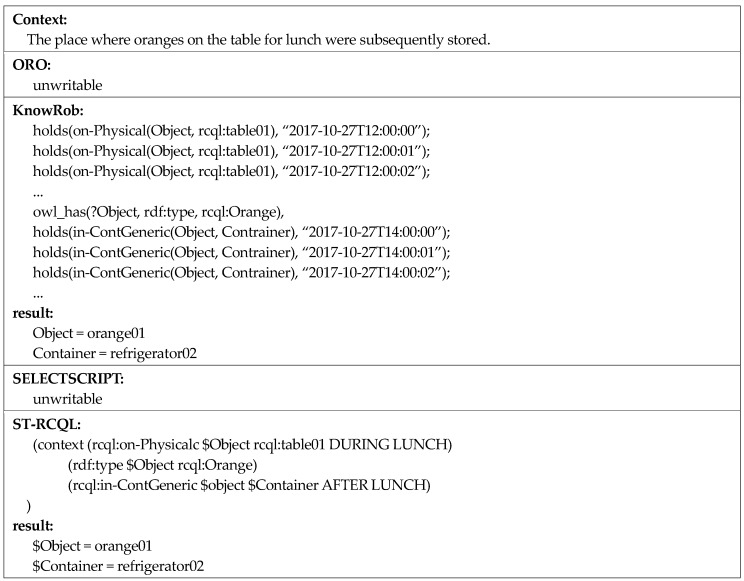
Comparison of spatio-temporal context queries three.

Compared to the first context, for the second context, “objects that were on the table yesterday,” the time point of the queried context is in the past and not the present. As mentioned earlier, ORO and SELECTSCRIPT do not maintain past context knowledge, and thus cannot write queries for a past context owing to the limitation of query grammar. Although KnowRob can write the query, as it only supports the time-point operator at, a very inefficient query is written to express the time interval of yesterday. In contrast, as ST-RCQL supports time-interval operators, it can write a very concise, efficient query, as shown in Figure 17. To verify the high linguistic expressive power of ST-RCQL, a query about a spatio-temporal context, in which various time points of the past and present are entangled, was written, as shown in the following example (Figure 18).

The third context is “The place where oranges on the table for lunch were subsequently stored.” To determine this context, multiple queries were constructed to find the oranges present on the table during lunch and then to find the place where they were stored after lunch. As in the second context, this third context also includes a past time point. Thus, ORO and SELECTSCRIPT cannot be used to write a query for this context. Although KnowRob can be used, it is still very inefficient. ST-RCQL can be used to write this query in only three lines. The first line finds the objects that were on the table, the second line selects oranges only among the objects present on the table at that time, and the third line finds the place where oranges were stored after lunch.

**Figure 19:**
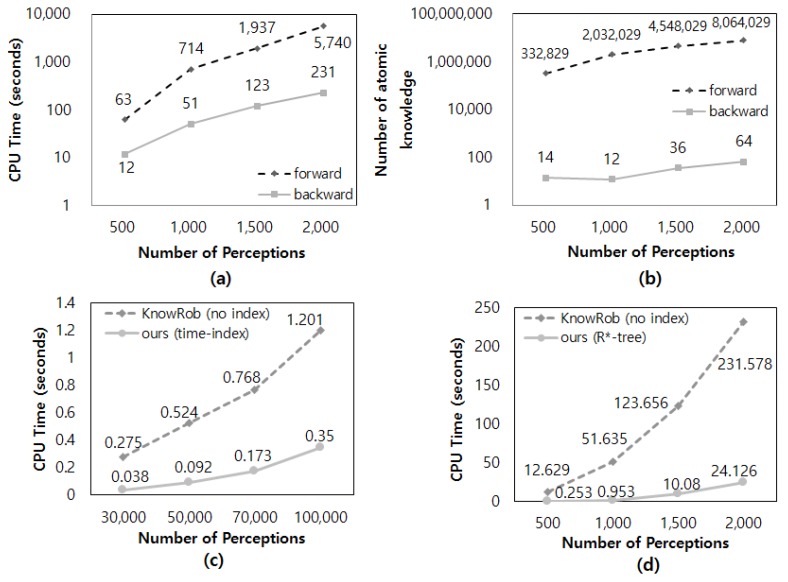
Experimental result of context query-processing by using reasoning types or spatio-temporal index (**a**) Reasoning time by reasoning types, (**b**) amount of inferred knowledge by reasoning types, (**c**) temporal query-processing time and (**d**) spatial query-processing time with respect to index.

Next, quantitative experiments were conducted to verify the efficiency of query-processing based on backward reasoning adopted in this study (Figure 19a,b) and the acceleration of query-processing by using spatio-temporal indices (Figure 19c,d).

Figure 19a shows the reasoning times of forward and backward reasoning depending on the number of visual perception instances stored in the working memory. All the visual perception instances were assumed to have the same valid time. Unlike backward reasoning, forward reasoning infers all possible context knowledge before querying; this requires considerable reasoning time. Furthermore, we determined that the reasoning time of forward reasoning increased exponentially with the number of visual perception instances. Figure 19b shows the volume of context knowledge inferred after forward and backward reasoning, depending on the number of visual perception instances stored in the working memory. Backward reasoning derives a very small volume of context knowledge, which it derives through reasoning among the inferred context knowledge by accessing specific context knowledge only. In contrast, forward reasoning derives a very large volume of context knowledge because it infers all possible knowledge and stores all the results. This results in an exponential increase in the volume of context knowledge with the increase in visual perception instances. In Figure 19b, the space occupied by approximately eight million triples of context knowledge in the memory, which was derived at the maximum, is approximately 1.5 GB. Visual perception instances obtained using the RGB-D camera are generated at the rate of approximately 10 frames per second. Therefore, approximately three minutes and 20 seconds is required to occupy 1.5 GB of memory. Furthermore, robots also obtain other perception information in addition to visual perception instances, and thus the memory capacity for forward reasoning is practically impossible to meet. Although not shown in the results of the two experiments in Figure 19a,b, to actually apply forward reasoning to robots, it must be performed ceaselessly whenever the perception occurs. This reasoning method is inappropriate for service robots that must work in real time.

Next, Figure 19c shows the temporal query-processing time according to the number of visual perception instances stored in the working memory. Figure 19c shows the graph of temporal query-processing times with and without the time index. For temporal query, the temporal query predicates in Figure 14 and Figure 15 were used. As temporal queries are extremely fast to process, the number of visual perception instances was increased more than in other experiments in Figure 19 to show a clear difference. The results in Figure 19c confirm that the use of a time index greatly accelerated the query-processing speed compared to the case of not using the time index. The effect of the index appears more conspicuously in the result of Figure 19d, which shows the spatial query-processing time depending on the number of visual perception instances stored in the working memory. Figure 19d shows the spatial query-processing times with and without the spatial index R*-tree. For a spatial query, the *owl_has* query predicate and the computable spatial predicate were used. The result in Figure 19d confirms that the spatial query-processing speed accelerated significantly compared to the case where the index was not used. Furthermore, the effect increased continuously with the number of visual perception instances.

## 7. Conclusions

In this paper, we proposed the context query language ST-RCQL and query-processing system ST-RCQP for service robots working in an indoor environment. The proposed context query language ST-RCQL was designed to query 3D spatial relations among objects at various periods based on Allen’s interval algebra. Furthermore, the automatic query-translation rules were designed to write very concise and intuitive queries by considering the nature of service robots, which mainly handle abstracted symbolic knowledge in terms of service. Furthermore, to support the real-time property of service robots, this study proposed a query-processing method of backward reasoning and a method of accelerating query-processing by the building of spatio-temporal indices for the individual perception information of objects. The suitability of ST-RCQL as a robot context query language and the efficient performance of ST-RCQP were verified through various experiments.

From the perspective of storing and retrieving context knowledge, one of the problems that must be dealt with as much care as time dependence is uncertainty, which was not addressed in this study. In the future, we plan to research a context query language and processing method that considers both time dependence and uncertainty of a context language.

## Figures and Tables

**Table 1:** Rules of query translation.

Query Pattern	Translation to Prolog Query
(context (SpatialPredicate $Subject $Object))	rdfs_individual_of(Subject, rcql:’SpatialThing’),
	rdfs_individual_of(Object, rcql:’SpatialThing’),

	latest_detection_of_instance(Subject, LatestDetectionS),
	latest_detection_of_instance(Object, LatestDetectionO),

	rdf_triple(SpatialPredicate, LatestDetectionS, LatestDetectionO).
(context (SpatialPredicate $Subject $Object “TimePointOperator” “TimePoint”))	rdfs_individual_of(Subject, rcql:’SpatialThing’),
	rdfs_individual_of(Object, rcql:’SpatialThing’),

	rdf_triple(knowrob:’objectActedOn’, SP, Subject),
	rdf_triple(knowrob:’objectActedOn’, OP, Object),

	time_point_operation(TimePointOperator, TimePoint, SP, OP),
	rdf_triple(SpatialPredicate, SP, OP).
(context (SpatialProperty $Subject $Object “TimeIntervalOperator” “TimePointS” “TimePointE”))	rdfs_individual_of(Subject, rcql:’SpatialThing’),
	rdfs_individual_of(Object, rcql:’SpatialThing’),

	rdf_triple(knowrob:’objectActedOn’, SP, Subject),
	rdf_triple(knowrob:’objectActedOn’, OP, Object),

	time_interval_operation (TimeIntervalOperator, TimePointS, TimePointE, SP, OP),
	rdf_triple(SpatialPredicate, SP, OP).
